# H_2_S biogenesis by cystathionine beta-synthase: mechanism of inhibition by aminooxyacetic acid and unexpected role of serine

**DOI:** 10.1007/s00018-022-04479-9

**Published:** 2022-07-21

**Authors:** Maria Petrosino, Karim Zuhra, Jola Kopec, Andrew Hutchin, Csaba Szabo, Tomas Majtan

**Affiliations:** 1 Department of Pharmacology, Faculty of Science and Medicine, University of Fribourg, Chemin du Musee 18, PER17, 1700 Fribourg, Switzerland; 2 Structural Biology Unit, Evotec Ltd, 114 Innovation Drive, Abingdon, OX14 4RZ UK

**Keywords:** Pyridoxal phosphate, Hydrogen sulfide, Enzyme kinetics, Homocystinuria, Cancer, Down syndrome

## Abstract

Cystathionine beta-synthase (CBS) is a pivotal enzyme of the transsulfuration pathway responsible for diverting homocysteine to the biosynthesis of cysteine and production of hydrogen sulfide (H_2_S). Aberrant upregulation of CBS and overproduction of H_2_S contribute to pathophysiology of several diseases including cancer and Down syndrome. Therefore, pharmacological CBS inhibition has emerged as a prospective therapeutic approach. Here, we characterized binding and inhibitory mechanism of aminooxyacetic acid (AOAA), the most commonly used CBS inhibitor. We found that AOAA binds CBS tighter than its respective substrates and forms a dead-end PLP-bound intermediate featuring an oxime bond. Surprisingly, serine, but not cysteine, replaced AOAA from CBS and formed an aminoacrylate reaction intermediate, which allowed for the continuation of the catalytic cycle. Indeed, serine rescued and essentially normalized the enzymatic activity of AOAA-inhibited CBS. Cellular studies confirmed that AOAA decreased H_2_S production and bioenergetics, while additional serine rescued CBS activity, H_2_S production and mitochondrial function. The crystal structure of AOAA-bound human CBS showed a lack of hydrogen bonding with residues G305 and Y308, found in the serine-bound model. Thus, AOAA-inhibited CBS could be reactivated by serine. This difference may be important in a cellular environment in multiple pathophysiological conditions and may modulate the CBS-inhibitory activity of AOAA. In addition, our results demonstrate additional complexities of using AOAA as a CBS-specific inhibitor of H_2_S biogenesis and point to the urgent need to develop a potent, selective and specific pharmacological CBS inhibitor.

## Introduction

The transsulfuration pathway represents the only route for de novo biosynthesis of cysteine in mammals and plays a central role in sulfur metabolism and redox regulation [1]. The pathway consists of two enzymatic steps. In the first step, cystathionine beta-synthase (CBS) irreversibly diverts sulfur from the methionine cycle by condensing homocysteine (Hcy) with serine (Ser) into cystathionine (Cth). In the second step, cystathionine gamma-lyase (CSE) hydrolyses Cth into cysteine (Cys), which is essential for protein synthesis, biosynthesis of glutathione and taurine, redox regulation and biogenesis of the gaseous signaling molecule hydrogen sulfide (H_2_S) [2]. Both CBS and CSE are pyridoxal-5′-phosphate (PLP)-dependent enzymes, which are responsible for the majority of H_2_S biosynthesis. Lacking strict substrate- and reaction-specificity, CBS and CSE catalyze H_2_S-generating reactions utilizing Hcy and Cys [3–5]. However, the regulatory aspects of CBS substrate preference and alternative reactivity are still poorly understood.

The pivotal role of CBS in the control of Hcy and H_2_S metabolism gives this enzyme an important pathophysiological relevance. Lack of CBS activity results in classical homocystinuria, an inborn error of metabolism characterized by highly elevated plasma and tissue concentrations of Hcy. Accumulation of Hcy results in ocular, skeletal and connective tissue abnormalities, mental retardation, thromboembolic events and stroke [6]. CBS-deficient homocystinuria is chiefly caused by pathogenic missense mutations leading to conformational instability, misfolding and ultimately degradation of CBS. Studies using proteasome inhibitors illustrated that misfolding defects of certain CBS mutants can be rescued by their refolding [7]. Therefore, it has been proposed that reversible competitive CBS inhibitors might act as pharmacological chaperones and rescue proper folding of certain CBS mutants [8]. On the other end of the CBS activity scale, increased expression of CBS results in an increased biogenesis of H_2_S. Upregulation of CBS and consequent overproduction of H_2_S stimulate mitochondrial electron transport, enhance cellular bioenergetics and increase proliferation in multiple types of cancer [9–11]. However, systemic and chronic overexpression of CBS due to additional copy of CBS gene in trisomy 21 (Down syndrome, DS) elevates the cellular levels of H_2_S to toxic levels, which (on the background of a variety of additional biochemical misalignments caused by additional gene triplications in this condition) suppresses mitochondrial Complex IV activity and impairs mitochondrial oxygen consumption and aerobic ATP generation [12]. Inhibition of CBS exerts suppression of cancer cell functions, while in DS, it results in normalization of H_2_S biogenesis and mitochondrial function [9, 12]. Thus, although the underlying mechanisms are markedly different, CBS inhibition emerges in both cancer and DS as a potential experimental therapeutic intervention.

In spite of multiple small-molecule screening campaigns seeking to discover novel, potent and selective CBS inhibitors [13–15], aminooxyacetic acid (AOAA) remains one of the most potent and widely used CBS inhibitor to date, even though, clearly, it also inhibits a variety of other enzymes including CSE and several transaminases [2]. AOAA belongs to a group of hydroxylamine compounds, which have marked reactivity against ketones and aldehydes. Previous studies suggested that the mechanism of action of AOAA involves attack on a Schiff base linkage on an internal aldimine between the PLP cofactor and enzyme, forming an oxime type complex, thus acting as a suicide inhibitor and preventing PLP regeneration in the catalytic cycle as shown for CSE [16]. The recent crystal structure of another PLP-dependent enzyme human kynurenine aminotransferase-I complexed with AOAA supports this mechanism of inhibition [17]. An alternative mechanism of AOAA yielding inhibited enzyme may involve formation of a catalytically inactive form of PLP cofactor pyridoxamine-5’-phosphate (PMP), as shown for yeast CBS complexed with hydrazine type inhibitor [18]. Instead of an expected irreversible dead-end complex featuring a tightly bound PLP-inhibitor hydrazone, the enzyme turned over the hydrazine inhibitor and yielded a catalytically inactive pyridoxamine form. Molecular interactions of AOAA with CBS have so far not been elucidated in detail apart from the docking simulation of CBS and PLP-AOAA complex [19].

To gain better understanding of the inhibitory mechanism of action of AOAA on CBS, we performed spectroscopic, biochemical, cellular and crystallographic studies with a goal to characterize the binding of AOAA to CBS and its reversibility and competition with CBS substrates. The results presented in the current report challenge the commonly held view of AOAA being an irreversible CBS inhibitor and may have profound consequences on future use of AOAA as CBS inhibitor and interpretation of cellular or in vivo studies.

## Materials and methods

*CBS enzymes.* Recombinant truncated *Saccharomyces cerevisiae* CBS lacking the C-terminal regulatory domain (residues 346–507) and carrying the C-terminal 6xHis tag (tScCBS) was prepared as described previously [20]. Engineered recombinant *Homo sapiens* CBS lacking a loop protruding from the central β-strand of the CBS2 domain corresponding to the residues 516–525 (HsCBS; described in detail in [21]) was purchased from GenScript.

*UV − Vis and fluorescence spectroscopy*. Absorption spectra were collected on a Tecan Infinite M200 Pro spectrophotometer in a buffer containing 50 mM Tris–HCl pH 8.5, 20 mM NaCl and 0.5 mM tris(2-carboxyethyl)phosphine (TCEP). All the spectra were carried out with a protein concentration of 1 mg/mL with a 1 cm path-length quartz cuvette in a total volume of 150 µL. tScCBS was titrated directly in a cuvette by successive additions of different concentrations of AOAA, Ser and Cys, respectively, and spectra were recorded between 240 and 600 nm after an incubation time of 3 min at RT. Fluorescence emission spectra were collected under the same conditions upon PLP excitation at 410 nm. Blank spectra were subtracted from sample spectra.

*Circular dichroism (CD) spectroscopy.* CD spectra were recorded on a Jasco J-1500 CD spectropolarimeter equipped with a Peltier-type temperature controller. Near-UV (250–450 nm) CD spectra of 1 mg/mL tScCBS were collected at 25°C in 1 cm path-length quartz cuvette at a scan rate of 50 nm/min in a buffer containing 50 mM Tris–HCl pH 8.5, 200 mM NaCl and 200 µM TCEP. Far-UV (190–250 nm) CD spectra of 0.1 mg/mL tScCBS were collected in a 0.1 cm path-length quartz cuvette in the same buffer. A minimum of three accumulations were made for each scan, averaged and corrected for the blank solution of the corresponding buffer. The results were expressed as the mean residue ellipticity ([Θ]), assuming a mean residue molecular mass of 110 per amino acid residue.

*CBS coupled-coupled assay.* CBS canonical activity utilizing Ser and Hcy was determined using cystathionine beta-lyase (CBL) and lactate-dehydrogenase (LDH) coupled-coupled assay as described previously [22] with a few modifications. Native L-LDH from rabbit muscle was purchased from Sigma (catalog no. L2500) and recombinant CBL was purchased from Creative Enzymes (catalog no. NATE-1146). In a final volume of 150 µL, the reaction mixture contained 50 mM Tris–HCl pH 8.9, 20 µM PLP, 500 µM NADH, 1.3 µM LDH, 0.1 µM CBL, 0–20 mM Ser (or 0–50 mM Cys) and 500 ng of tScCBS or HsCBS. Assays with HsCBS were carried out in the absence or presence of 500 µM CBS allosteric activator S-adenosylmethionine (SAM). The reaction was triggered by the addition of 2 mM Hcy and the oxidation of NADH to NAD^+^ was followed by monitoring the decrease in absorbance at 340 nm over time (ε_340_ = 6220 M^−1^.cm^−1^). Since Ser and Cys serve as inferior CBL substrates compared to CBS-generated Cth [23], possible assay interference was determined by carrying out the assay for each Ser (or Cys) concentration in the absence of CBS. All measurements were performed in triplicates using Tecan Infinite 200 PRO microplate reader. Kinetic data were analyzed using GraphPad Prism software.

*CBS H*_*2*_*S production assay.* H_2_S-producing activity of CBS, utilizing Cys and Hcy, was determined using a fluorometric assay employing the H_2_S-selective fluorescent probe 7-azido-4-methylcoumarin (AzMC) as described previously [14]. The reaction mixture in a total volume of 200 µL contained 50 mM Tris–HCl pH 8.6, 5 μM PLP, 2 mM Hcy, 10 μM AzMC and 500 ng tScCBS or HsCBS. The plate was incubated for 10 min at 37°C and the enzymatic activity was triggered by adding 0–50 mM Cys. The fluorescence of the mixtures at 450 nm (excited at 365 nm) was followed for 2 h at 37°C. All measurements were performed in triplicates using Tecan Infinite 200 PRO microplate reader. Kinetic data were analyzed using GraphPad Prism software.

*Cell culture.* The human embryonic kidney cell line HEK293 (ATCC CRL-1573) was cultured in Dulbecco’s Modified Eagle Medium (DMEM) containing 4.5 g/L glucose. The culture medium was supplemented with 10% (v/v) heat-inactivated fetal bovine serum, 2 mM Glutamax, non-essential amino acids, 100 U/ml penicillin and 100 μg/ml streptomycin. Cells were grown in a humidified incubator at 37 °C and 5% CO_2_ atmosphere. Cell viability and proliferation were assessed using MTT and BrdU assay, respectively, essentially as described previously [24].

*Detection of H*_*2*_*S production in live cells.* Cells were seeded in black 96-well plates with optical bottom (15,000 cells/well). The following morning, the cells were treated with 150 µM AOAA, 1/3/10 mM Ser or their combination for 24 h. After that the culture medium was replaced with Hank’s Balanced Salt Solution (HBSS) buffer supplemented with 100 μM AzMC and further incubated for 1 h. Dye’s specific fluorescence was visualized using a Leica DFC360 FX microscope and images were captured with Leica Application Suite X software (Leica Biosystems, Germany). Images were analyzed with ImageJ software (version 1.8.0; NIH, Bethesda, MD, USA) and data were analyzed using GraphPad Prism 8 (GraphPad Software Inc.; San Diego, CA, USA).

*Bioenergetic analysis.* The Seahorse XFe24 flux analyzer (Agilent Technologies, Santa Clara, CA, USA) was used to estimate mitochondrial respiration of HEK293 cells. Cells were seeded in Seahorse XFe24 Cell Culture Microplates at density of 15,000 cells/well in a total volume of 200 µl, incubated for 2 h, followed by treatment for 24 h as described above for H_2_S live imaging. Next day, culture medium was replaced for Seahorse XF DMEM supplemented with 2 mM glutamine, 1 mM pyruvate and 10 mM glucose. The microplate was then incubated in a CO_2_-free incubator at 37°C for 1 h, to allow temperature and pH equilibration, as recommended by the manufacturer. The assay protocol included two measurements of basal values of oxygen consumption rate (OCR), followed by the injection of 1 µM oligomycin, used to evaluate ATP generation rate. Subsequently, the mitochondrial oxidative phosphorylation uncoupler FCCP (0.2 µM) was added to estimate maximal mitochondrial respiratory capacity. Lastly, 0.5 µM of rotenone and antimycin A (each) were injected to inhibit the electron flux through the complex I and III, respectively, aiming to detect the extra-mitochondrial OCR. Data were analyzed with Wave package (version 2.6; Agilent Technologies, Santa Clara, CA, USA) and GraphPad Prism 8 (GraphPad Software Inc.; San Diego, CA, USA).

*Statistical analysis.* Data are presented as mean ± standard error of the mean (SEM). Statistical analysis was conducted using t-test or ANOVA followed by Bonferroni’s multiple comparison test to determine significance designated by asterisks (*p < 0.05, **p < 0.01 and ***p < 0.001).

*Re-purification of HsCBS for crystallization trial.* Partially purified HsCBS from Genscript contained the N-terminal 6xHis tag followed by a TEV protease recognition site. Re-purification of HsCBS for crystallization involved removal of the 6xHis tag by in-house produced TEV protease followed by a reverse metal affinity chromatography. The sample obtained in the flow-through was diluted 10 × with 10 mM HEPES pH 7.5, 5% glycerol, 10 µM TCEP and loaded onto a 6 mL Cytiva ResourceQ anion exchange column equilibrated in Buffer A (50 mM HEPES pH 7.5, 50 mM NaCl, 5% glycerol, 10 µM TCEP). The protein was eluted using a 20 column volume gradient from 100% Buffer A to 100% Buffer B (50 mM HEPES pH 7.5, 1 M NaCl, 5% glycerol, 10 µM TCEP). Fractions containing HsCBS were pooled and loaded onto Cytiva HiLoad 16/600 Superdex 200 column equilibrated with 10 mM HEPES pH 7.5, 500 mM NaCl, 5% glycerol, 10 µM TCEP. The peak fractions were pooled and concentrated to 16 mg/ml and flash frozen in liquid nitrogen.

*Crystallization and data collection.* The re-purified HsCBS at 10 mg/mL with 1 mM AOAA was centrifuged (13,000 rpm, 15 min, 4°C) before establishing crystallization screens. Crystals grew at 4°C within 1 week in a solution containing 21% PEG 3350 and 140 mM sodium formate. Crystals were cryoprotected with 30% ethylene glycol and flash frozen in liquid nitrogen. X-ray diffraction data were collected at Diamond Light Source beamline I03 equipped with Eiger2 XE 16 M detector.

*Refinement.* Diffraction data were processed using autoPROC [25]. Crystals of HsCBS in complex with AOAA belonged to a space group *I222* (unit cell parameters: *a* = 125.6 Å, *b* = 134.6 Å, *c* = 169.3 Å, *α* = 90.0°, *β* = 90.0°, *γ* = 90.0°) and diffracted to ellipsoidal resolution limits of 2.70 Å in the a* direction, 4.51 Å in the b* direction and 2.69 Å in the c* direction, as determined by an anisotropic locally averaged signal/noise ratio of greater than 1.2, implemented by STARANISO [26]. Molecular replacement was performed using Phaser [27] and PDB model 4COO [28]. Cycles of restrained refinement and iterative model building were performed using Coot [29] and BUSTER [30]. Model quality was assessed using MolProbity [31]. Data collection and refinement statistics are presented in Table 1.

**Table 1:** Data collection and refinement

PDB ID	7QGT		
Ligand	PLP (chain A), AOAA-PLP (chain B)		
Data collection			
Beamline	Diamond Light Source I03		
Wavelength [Å]	0.97628		
Space group	I222		
Unit cell parameters [Å]	125.6	134.6	169.3
α = β = γ [°]	90		
	Overall	Inner shell	Outer shell
Resolution range [Å]	100–2.69	100–9.1	2.92–2.69
Total number of observations	260,805	14,713	13,972
Total number unique	22,433	1120	1123
Rmerge (%)	37.2	7.3	194
I/σ(I)	6.5	21.8	1.4
Completeness (spherical)	55.9	99.7	12.9
Completeness (ellipsoidal)	92.9	99.7	62.9
Multiplicity	11.6	13.1	12.4
CC(1/2)	0.988	0.998	0.668
Refinement			
R_work_ (%)	21.5		
R_free_ (%)	25.0		
Wilson Bfactor [Å^2^]	58.4		
Average total B factor (Å^2^)	48.3 (Chain A)		
	52.5 (Chain B)		
Average ligand B factor (Å^2^)	35.0 (Chain A)		
	42.2 (Chain B)		
R.m.s.d. bond length (Å)	0.008		
R.m.s.d. bond angle (°)	0.86		
Ramachandran outliers (%)	0		
Ramachandran favored (%)	97.3		

*Modeling.* A theoretical model of HsCBS with serine was prepared by superpositioning of the previously published CBS structures in complex with serine from *Drosophila melanogaster* DmCBS (PDB# 3PC4 [32]) and tScCBS (PDB# 6C2Q [18]) to the structure of HsCBS (PDB# 4COO [28]) and transferring the serine molecule to the structure of HsCBS, followed by energy minimization in MOE (Chemical Computing Group ULC). Figures were prepared using Chimera v1.15 [33] and PyMOL v2.3 (Schrodinger, USA).

## Results

### Spectroscopic characterization of CBS complexes with Ser, Cys and AOAA

The reaction intermediates of PLP with CBS substrates Ser or Cys as well as CBS inhibitor AOAA can be readily monitored by complementary absorption and fluorescence spectroscopy. Since human CBS contains an additional heme cofactor, which effectively masks absorption spectrum of PLP, for spectroscopic studies we utilized the heme-independent tScCBS lacking the regulatory CBS domain [20].

**Fig. 1:**
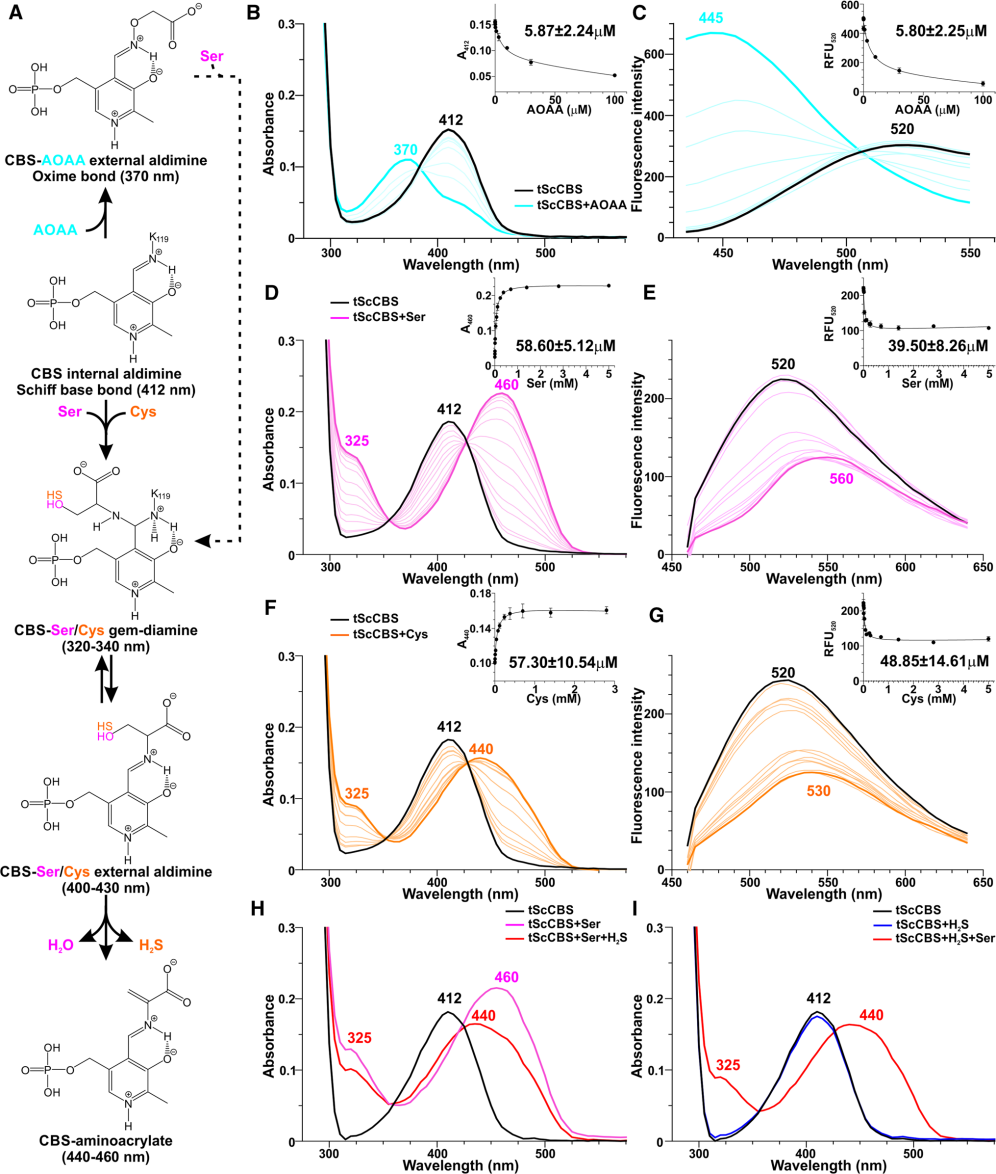
CBS reaction intermediates and UV–Vis absorption and fluorescence spectra of tScCBS in the presence of AOAA, Ser and Cys. **A** Proposed reaction mechanism of the first half of the CBS catalytic cycle with substrates (Ser, Cys) and inhibitor (AOAA) showing absorption maxima associated with the PLP-bound intermediates. **B**–**G** Changes in the UV–Vis absorption and fluorescence spectra of tScCBS (1 mg/mL in 50 mM Tris–HCl pH 8.5, 20 mM NaCl, 500 µM TCEP) upon addition of 0–100 µM AOAA (**B**–**C**), 0–5 mM Ser (**D**–**E**) and 0–5 mM Cys (**F**–**G**). Spectra were recorded after 3 min of incubation for each concentration. Fluorescence spectra were recorded between 435 and 650 nm after excitation at 410 nm. The insets show fittings of the spectral changes for the calculation of dissociation constants K_d_ from three independent measurements. **H**–**I** Changes in the UV–Vis absorption spectra of tScCBS upon addition of 1 mM Ser followed by addition of equimolar H_2_S donor (1 mM Na_2_S; **H**) and upon addition of 1 mM H_2_S followed by addition of equimolar 1 mM Ser (**I**)

Figure 1 shows CBS catalytic intermediates (Fig. 1A) and spectroscopic changes that occurred in the absorption and fluorescence profiles of tScCBS upon concentration-dependent titrations using AOAA, Ser and Cys (Fig. 1B–G, respectively). The UV–Vis spectrum of tScCBS showed a peak at 412 nm, corresponding to the internal aldimine formed between PLP and K53 residue (Fig. 1A, B, D, F). Titration with AOAA resulted in a blue-shift of the PLP peak to 370 nm, likely corresponding to an oxime PLP-AOAA intermediate (Fig. 1B). A dissociation constant K_d_ for AOAA of ~ 6 µM was determined by plotting the decrease in absorbance at 412 nm as a function of AOAA concentration (Fig. 1B inset). In contrast, titrations of tScCBS with Ser and Cys resulted in a red-shift of the PLP peak to 460 and 440 nm, respectively (Fig. 1D, F), corresponding to respective PLP-aminoacrylate intermediates. Dissociation constants K_d_ for Ser and Cys were determined by plotting the increase in absorbance at 460 and 440 nm as a function of Ser and Cys concentrations, respectively (Fig. 1D, F insets), and were approximately tenfold higher than for AOAA, i.e. ~ 58 µM. The presence of two isosbestic points at 355/353 nm and 426/430 nm, respectively (Fig. 1D, F), reflects the equilibrium between the gem-diamine and external aldimine intermediates.

Fluorescence spectroscopy represents a powerful complementary technique to UV–Vis spectroscopy when used to further characterize PLP reaction intermediates and isoforms [34]. Indeed, using the maximal PLP ketoenamine excitation wavelength of 410 nm, the emission spectrum of the PLP internal aldimine was centered at 520 nm (Fig. 1C, E, G). The binding of AOAA induced a 75 nm blue-shift to 445 nm along with an increase in the fluorescence intensity reflecting a lengthening of the ketoenamine excited-state lifetime and formation of PLP-AOAA oxime intermediate (Fig. 1C). In contrast, titration of tScCBS with Ser was accompanied by a 40 nm red-shift to 560 nm and decrease in fluorescence, both indicative of the first half of the catalytic cycle forming PLP-aminoacrylate intermediate (Fig. 1E). Similarly, titrations of tScCBS with Cys was accompanied by a 10 nm red-shift to 530 nm and decrease in fluorescence indicating PLP-aminoacrylate adduct (Fig. 1G). Dissociation constants K_d_ were determined by plotting the decrease in emission fluorescence at 520 nm as a function of concentration of the studied compounds and were comparable to those obtained using UV–Vis spectroscopy (Fig. 1C, E, G insets).

The 20 nm difference in a position of the PLP-aminoacrylate peak upon binding of Ser (460 nm; Fig. 1D) and Cys (440 nm; Fig. 1F) was unexpected as the PLP-aminoacrylate intermediate is identical for both substrates. Since H_2_S is the leaving group eliminated from Cys to form aminoacrylate compared to water in case of Ser, we investigated the possibility that H_2_S was not fully dissociated from the enzyme and affected spectral properties of the PLP-aminoacrylate intermediate. Indeed, Fig. 1H shows that tScSCB treated with 1 mM Ser yielded the expected PLP-aminoacrylate with the absorption peak at 460 nm, which blue-shifted to 440 nm upon addition of the equimolar concentration of H_2_S. Similarly, while addition of 1 mM H_2_S did not elicit any spectral changes to the PLP of tScCBS, subsequent addition of 1 mM Ser resulted in a formation of the PLP-aminoacrylate absorbing at 440 nm (Fig. 1I), which is spectrally similar to the one produced in the presence of Cys (Fig. 1F).

Taken together, UV–Vis and fluorescence spectroscopic titrations suggest that AOAA binds tighter to PLP of tScCBS than the substrates, forming oxime adduct unlike substrates yielding aminoacrylate intermediates. The PLP-aminoacrylate intermediates from Ser and Cys are distinct, because the eliminated H_2_S from Cys remains associated with the catalytic center and interferes with the spectral properties of PLP-aminoacrylate species.

### Reversibility of the CBS-inhibitory effect by AOAA

**Fig. 2:**
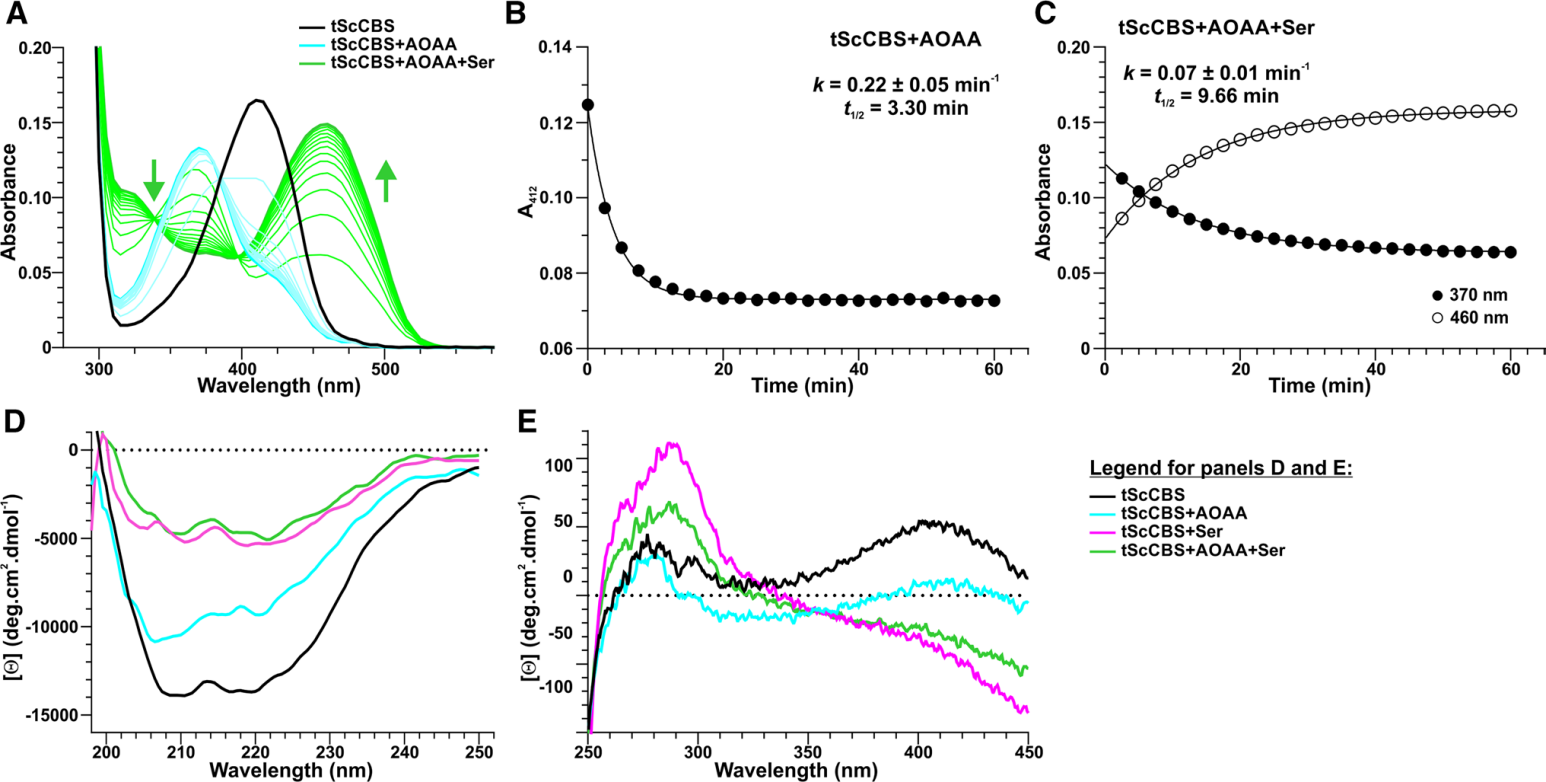
Spectroscopic rescue of AOAA-inhibited tScCBS by Ser. **A** Changes in the absorption spectra of tScCBS upon addition of 20 µM AOAA (cyan lines) followed for 1 h. Subsequently, 1 mM Ser (gray lines) was added and spectral changes were followed for additional 1 h. **B** Representative kinetic fitting of AOAA binding to tScCBS followed at 412 nm. **C** Representative kinetic fitting of AOAA displacement from tScCBS by Ser by following changes at 370 nm and 460 nm. The kinetic parameters of the observed processes are shown as insets in panels **B** and **C**. Far-UV (**D**) and near-UV (**E**) CD spectra of tScCBS (black), tScCBS complexed with 20 µM AOAA (cyan) or 1 mM Ser (magenta) and tScCBS complexed with 20 µM AOAA followed by 1 h incubation with 1 mM Ser (green). All spectra were recorded at 25 °C

Based on the action of AOAA on other PLP-dependent enzymes, it has been assumed that AOAA acts as an irreversible dead-end suicide inhibitor of CBS [16, 17]. However, our data show that Ser, a canonical CBS substrate, can displace AOAA from the PLP site (Fig. 2). Addition of 20 µM AOAA to tScCBS (corresponding to twofold excess compared to IC_50_; Fig. 4A) resulted in a fast formation of PLP-AOAA oxime intermediate with a rate constant *k* of 0.22 min^−1^ (Fig. 2A, B). The subsequent addition of 1 mM Ser induced an approximately 3 × slower formation of PLP-aminoacrylate intermediate, as indicated by a red-shift of the PLP-AOAA peak at 370 nm to PLP-aminoacrylate peak at 460 nm (Fig. 2A, C). Interestingly, the displacement of AOAA from the tScCBS active site did not occur in the presence of Cys (data not shown) suggesting that only the canonical substrate is able to revert the inhibition of CBS.

To confirm our unexpected observation that Ser can indeed displace AOAA and thus reactivate tScCBS, we next employed CD spectroscopy to investigate the potential conformational and structural changes in tScCBS associated with binding of AOAA and Ser (Fig. 2D, E). The far-UV CD (190–250 nm) spectrum of the native tScCBS showed the two strong negative contributions at 208 nm and 222 nm, typical of proteins with high percentage of alpha helices (Fig. 2D). The far-UV CD spectrum of tScCBS complexed with AOAA showed a similar shape with moderate (~ 25%) changes in the molar ellipticity intensity. However, a strong decrease in the negative molar ellipticity (~ 65%) of the minima at 208 nm and 222 nm was observed in the spectra of both tScCBS complexed with Ser and tScCBS complexed with AOAA followed by 1 h incubation with Ser (Fig. 2D). Furthermore, the 222/208 molar ellipticity ratio, which is indicative of interhelical contacts present in helix bundle and coiled-coil structures, differed in the analyzed samples and corresponded to 0.94, 0.88, 1.11 and 1.41 in tScCBS, tScCBS complexed with AOAA, tScCBS conjugated with Ser and tScCBS complexed with AOAA and incubated for 1 h with Ser, respectively. The near-UV (250–450 nm) CD spectrum of the native tScCBS exhibited a pronounced positive peak at 410 nm and a lower positive band around 280 nm (Fig. 2E). Addition of 20 µM AOAA resulted in a decrease in the molar ellipticity at 410 nm and a reduction of the 280 nm band while preserving the same profile as tScCBS. In contrast, Ser induced a complete disappearance of the 410 nm peak and a marked increase of the 280 nm band. By comparing the UV–Vis spectrum and the near-UV CD spectrum of tScCBS complexed with Ser, we attribute the strong negative contribution at 450 nm to the PLP-aminoacrylate intermediate. Finally, tScCBS complexed with AOAA followed by 1 h incubation with Ser showed similar near-UV CD spectrum as tScCBS complexed with Ser.

Together, our UV–Vis and CD spectroscopy studies show that the CBS inhibitor AOAA can be replaced by Ser, indicating that AOAA inhibition of CBS is reversible.

### Steady‑state characterization of CBS

**Fig. 3:**
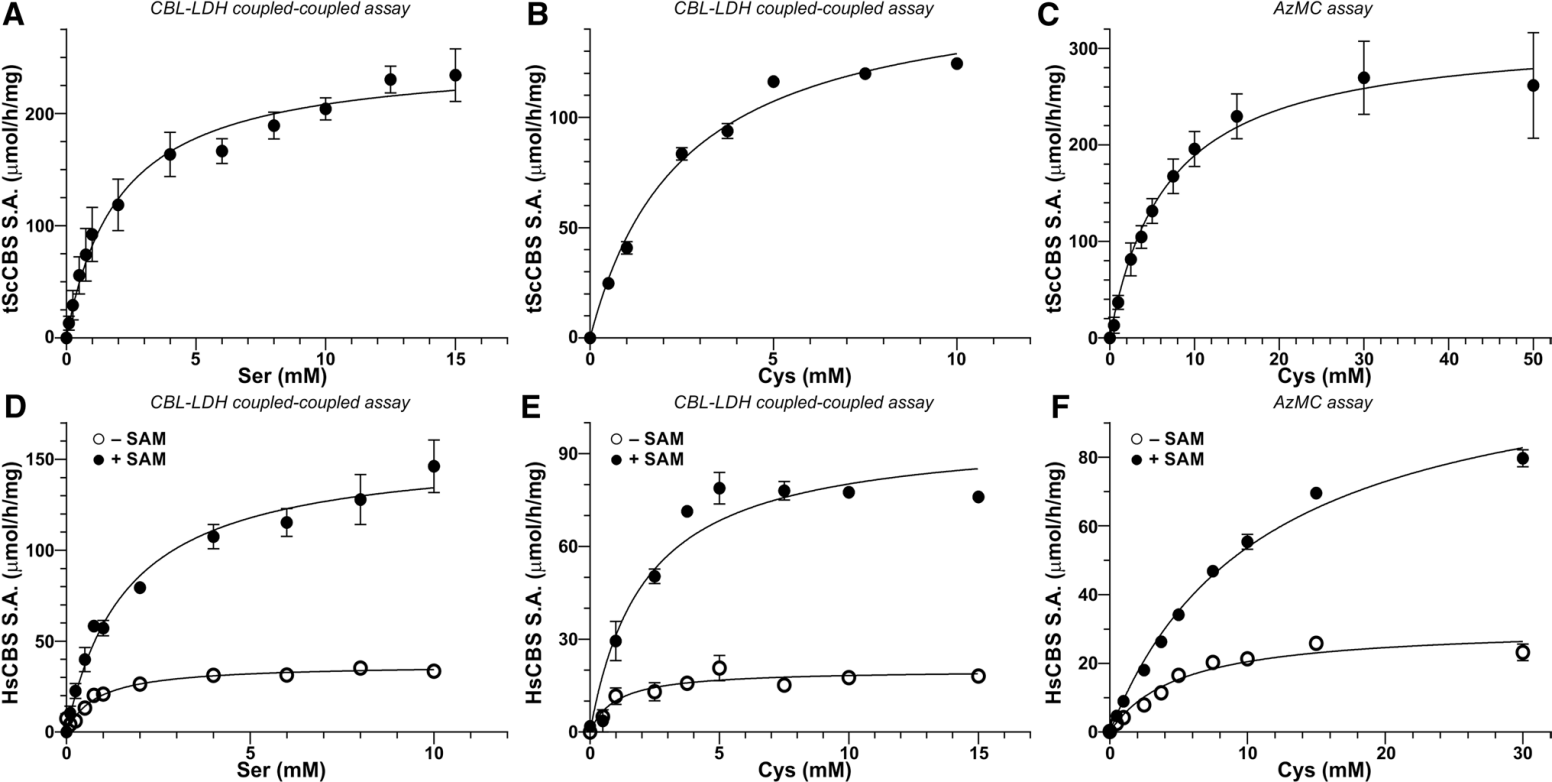
Enzyme kinetics. Steady-state enzyme kinetics was performed for tScCBS (**A–C**) and HsCBS in the absence and presence of 500 µM SAM (**D**–**F**). Enzyme kinetics for Ser was executed using CBL-LDH coupled-coupled assay (**A, D**), while both the CBL-LDH coupled-coupled and AzMC assays were used for determining kinetics parameters for the alternative substrate Cys (**B, C, E, F**). Data were fitted using the Michaelis–Menten equation with K_m_ and V_max_ determined directly from the non-linear fit. These parameters along with other calculated ones are summarized in Table 2

To confirm the functional consequences of our spectroscopic observations that CBS inhibition by AOAA can be rescued by Ser, but not Cys, we determined steady-state enzyme kinetics for both tScCBS and HsCBS in the canonical and alternative H_2_S-generating reaction as well as characterized CBS activity in the presence of AOAA. Figure 3 shows enzyme kinetics assays for tScCBS (Fig. 3A–C) and HsCBS in the absence and presence of SAM (Fig. 3D–F), while Table 2 summarizes all of the determined parameters. Use of a CBL-LDH coupled-coupled assay allowed us to determine kinetic parameters for the canonical Ser + Hcy and alternative Cys + Hcy condensations using the same assay and thus to compare them side-by-side. While K_m_s for Ser and Cys did not show substantial differences ranging from 0.8–2.5 mM, maximal velocities V_max_ were 36–46% lower for the alternative reaction compared to the canonical one. This translated into about 50% lower CBS efficiency for the biogenesis of H_2_S compared to the canonical reaction (Table 2). Interestingly, when we used a fluorometric AzMC assay to detect H_2_S production (as the product of Cys + Hcy condensation) instead of Cth determined using the CBL-LDH coupled-coupled assay, we observed substantial differences. Particularly, K_m_s for Cys were ~ 3–6 times higher while V_max_ remained similar or just slightly elevated, which translated in even lower enzyme efficiency to generate H_2_S compared to the canonical reaction (Table 2).

**Table 2:** Steady-state enzyme kinetics of tScCBS and HsCBS for both the canonical and the alternative H_2_S-generating reactions

Kinetic parameter	tScCBS	HsCBS – SAM	HsCBS + SAM
Ser + Hcy → Cth + H_2_O			
K_m_ (mM)	2.0 ± 0.4	0.8 ± 0.1	1.6 ± 0.2
V_max_ (μmol·h^−1^·mg^−1^)	250.7 ± 13.4	37.2 ± 1.5	156.3 ± 7.6
* k*cat (s^−1^)	2.7 ± 0.1	0.63 ± 0.06	2.7 ± 0.1
* k*cat/K_m_ (s^−1^·mM^−1^)	1.34	0.79	1.66
Cys + Hcy → Cth + H_2_S			
K_m_ (mM)^a^	2.5 ± 0.2	1.0 ± 0.4	2.1 ± 0.5
V_max_ (μmol·h^−1^·mg^−1^)^a^	161.5 ± 4.5	20.1 ± 1.7	97.2 ± 7.1
* k*cat (s^−1^)^a^	1.7 ± 0.1	0.34 ± 0.02	1.66 ± 0.03
* k*cat/K_m_ (s^−1^·mM^−1^)^a^	0.69	0.34	0.79
K_m_ (mM)^b^	6.7 ± 1.5	5.0 ± 0.9	11.1 ± 0.8
V_max_ (μmol·h^−1^·mg^−1^)^b^	317.0 ± 24.1	30.8 ± 1.9	113.3 ± 4.0
* k*cat (s^−1^)^b^	3.4 ± 0.3	0.52 ± 0.04	1.93 ± 0.06
* k*cat/K_m_ (s^−1^·mM^−1^)^b^	0.51	0.11	0.17

^a^CBL-LDH coupled-coupled assay

^b^AzMC assay

**Fig. 4:**
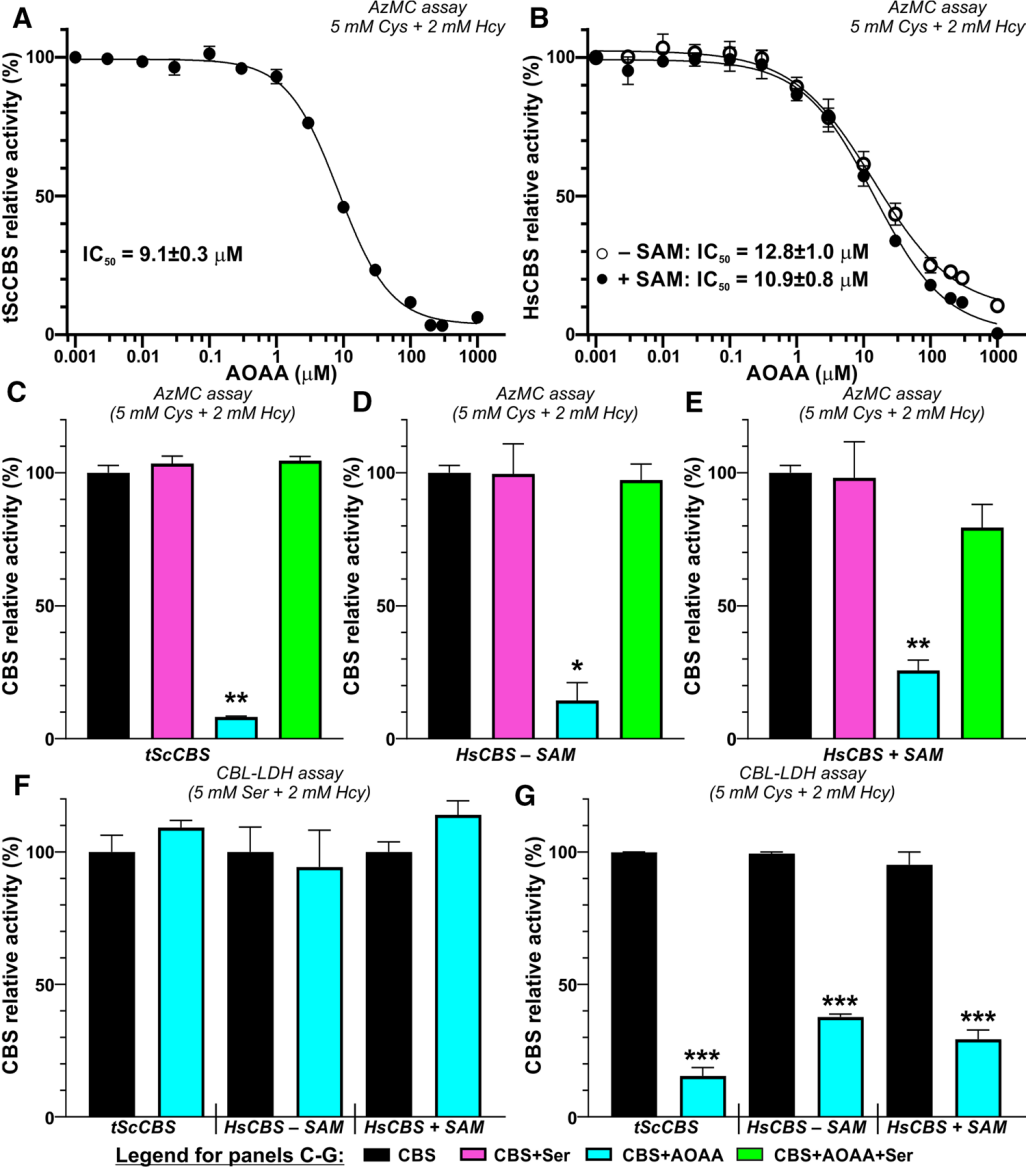
Functional rescue of AOAA-inhibited CBS by Ser. **A, B** Determination of IC_50_ of AOAA in both tScCBS (**A**) and HsCBS in the absence and presence of 500 µM SAM (**B**). The determined IC_50_ values from the best fit are shown. **C**–**E** Rescue of CBS activity by preincubation with 1 mM Ser (green) followed by AzMC assay (**C**–**E**) and CBL-LDH coupled-coupled assay in the presence of 5 mM Cys (or 5 mM Ser) and 2 mM Hcy from the enzyme inhibited by 200 µM AOAA (cyan) compared to untreated enzyme (black) or enzyme pretreated with 1 mM Ser (magenta). Panels **C**, **D** and **E** show the results for tScCBS, HsCBS in the absence and in the presence of 500 µM SAM, respectively. (**F**, **G**) Ser, but not Cys as a substrate rescued enzymatic activity of AOAA-inhibited tScCBS, HsCBS in the absence and in the presence of 500 µM SAM using the CBL-LDH coupled-coupled assay for both the canonical (5 mM Ser + 2 mM Hcy; **F**) and alternative reactivities (5 mM Ser + 2 mM Hcy; **G**)

Using the established fluorogenic H_2_S-detecting AzMC CBS activity assays, we first characterized potency of AOAA to inhibit CBS activity. The half maximal inhibitory concentration (IC_50_) of AOAA was determined as 9.1 µM for tScCBS (Fig. 4A) or 12.8 µM and 10.9 µM for HsCBS in the absence and presence of SAM, respectively (Fig. 4B). Incubation of tScCBS and HsCBS with 200 µM AOAA (~ 20-fold excess compared to IC_50_) followed by removal of unreacted excess of AOAA through a desalting column resulted in 75–92% inhibition of CBS activity suggesting that AOAA forms a stable adduct with PLP bound to CBS polypeptide (Fig. 4C–E). However, addition of 1 mM Ser after AOAA incubation with the enzyme followed by a desalting step resulted in a substantial recovery of tScCBS activity (Fig. 4C) as well as HsCBS activity in the absence and presence of SAM (Fig. 4D, E). Furthermore, we confirmed this observation using the CBL-LDH coupled-coupled assay, which allowed us to study the canonical (Ser + Hcy) and H_2_S-generating alternative (Cys + Hcy) reactions in the same system. While the condensation of Ser and Hcy was not impaired by AOAA (Fig. 4F), the activity of AOAA-inhibited tScCBS and HsCBS in the absence and presence of SAM catalyzing the condensation of Cys and Hcy yielding H_2_S was markedly reduced by 85, 63 and 71%, respectively (Fig. 4G).

Taken together, these results confirm our spectroscopic observations and suggest that AOAA selectively inhibits only the alternative H_2_S-producing activity of CBS and that AOAA-inhibited CBS can be reactivated by its canonical substrate Ser.

### Cellular consequences of AOAA-inhibited CBS reactivation by Ser

**Fig. 5:**
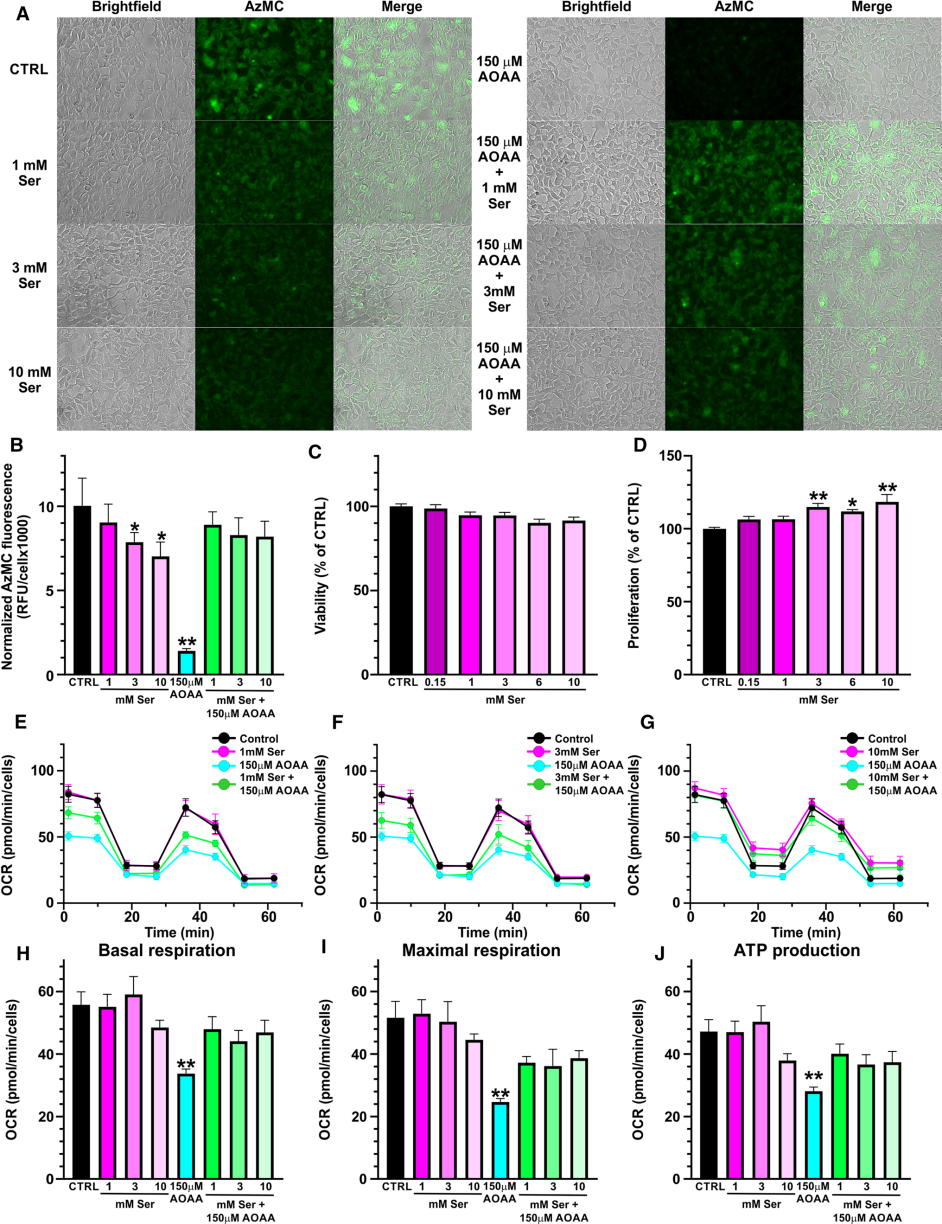
Rescue of AOAA-inhibited CBS-H_2_S activity by Ser in cellular context. **A** H_2_S production in HEK293 cells in response to 1/3/10 mM Ser, 150 µM AOAA or their combination was assessed by live fluorescent imaging using AzMC H_2_S-selective probe. **B** Quantification of the normalized cellular fluorescence shown in the representative pictures in panel **A**. **C**, **D**) Cell viability (**C**) and cell proliferation (**D**) of HEK293 cells after exposure to increasing concentrations of Ser (0.15–10 mM). **E**–**J** Cellular bioenergetics of HEK293 cells was assessed by Seahorse flux analyzer in the presence of increasing Ser concentrations 1 mM (**E**), 3 mM (**F**) and 10 mM (**G**). Basal respiration (**H**), maximal respiration (**I**) and ATP production (**J**) were calculated from the Seahorse flux data and compared. Data represents mean ± SEM values of at least five independent experiments

**Fig. 6:**
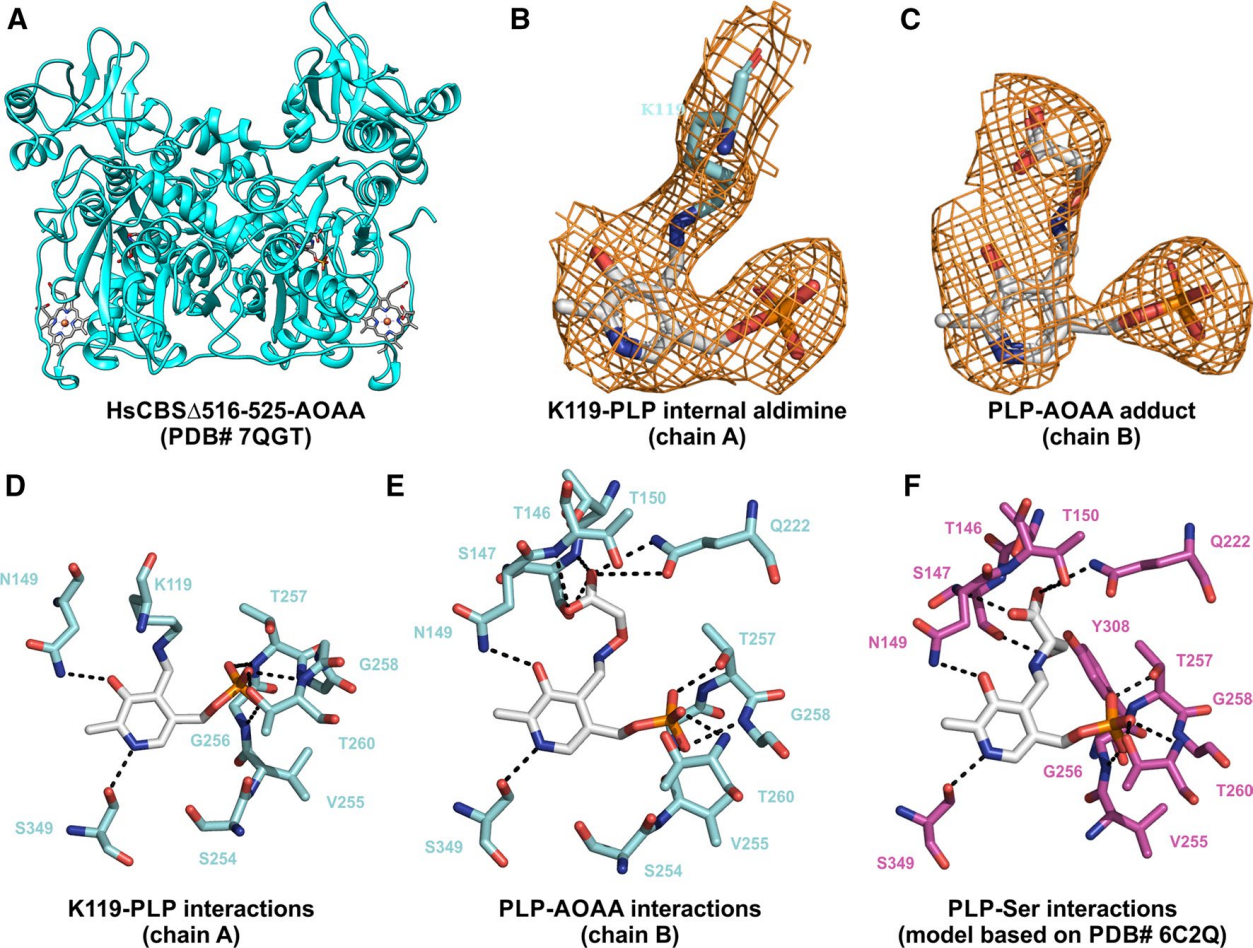
Structural insights into the inhibition of HsCBS by AOAA. **A** Overall fold and conformation of dimeric HsCBSΔ516-525 complexed with AOAA (cyan). **B, C** The Fo-Fc omit maps contoured at 1σ showing electron density around K119-PLP internal aldimine in chain A (**B**) and PLP-AOAA adduct in chain B of HsCBSΔ516-525 (**C**). **D, E** Residues of HsCBSΔ516-525 interacting with the K119-PLP internal aldimine (**D**) and PLP-AOAA adduct (**E**). **F** Model of HsCBSΔ516-525 with bound Ser based on crystal structure of tScCBS with Ser (PDB# 6C2Q [18]) showing interacting residues with PLP-Ser intermediate

Furthermore, we evaluated our spectroscopical and biochemical observation that Ser can reactivate AOAA-inhibited CBS in mammalian cells naturally expressing substantial amount of CBS, HEK293 (Fig. 5). As anticipated, HEK293 cells produced substantial amount of H_2_S, which was significantly reduced by up to ~ 30% after addition of Ser into medium in a concentration-dependent manner (Fig. 5A, B). Added Ser did not affect cell viability (Fig. 5C); however, cellular proliferation was slightly, but significantly increased only by the concentrations of Ser above 3 mM (up to ~ 18%; Fig. 5D). Addition of 150 µM AOAA resulted in a substantial decrease of H_2_S-producing activity of HEK293 cells to ~ 15% of the controls. More importantly, inhibition of CBS activity by 150 µM AOAA followed by addition of 1, 3 or 10 mM Ser resulted in a recovery of H_2_S-producing capacity of HEK293 cells achieving 82–89% of control WT activity (Fig. 6A, B). H_2_S production in HEK293 cells depends on CBS activity as knock-out of CBS resulted in a complete shutdown of a fluorescent signal from AzMC probe and therefore, function of Ser on H_2_S production could not have been tested in a CBS-independent manner (data not shown).

Changes in CBS activity and H_2_S production in response to AOAA and Ser were also confirmed following bioenergetics of HEK293 cells using Seahorse flux assay (Fig. 5E–J). Addition of 1, 3 and 10 mM Ser did not have any significant impact on mitochondrial function. On the other hand, 150 µM AOAA resulted in a significant decrease of all bioenergetics parameters, namely basal respiration (Fig. 5H), maximal respiration (Fig. 5I) and ATP production (Fig. 5J). However, supplementation of 1, 3 or 10 mM Ser to HEK293 cells inhibited by 150 µM AOAA resulted in a correction or normalization of cellular bioenergetics compared to the untreated controls.

Taken together, our cellular studies suggest that the reactivation of AOAA-inhibited CBS and its H_2_S-producing activity by Ser, conclusively confirmed by spectroscopical and biochemical techniques, also occurs in a cellular context, which may have significant consequences for the use of AOAA as pharmacological CBS-specific H_2_S inhibitor in vivo and interpretation of such data.

### Structural insight into HsCBS interaction with AOAA

In addition to the spectroscopic, catalytic and cellular consequences of AOAA binding to CBS, we solved the crystal structure of HsCBS in complex with AOAA to gain knowledge about its molecular interactions (Fig. 6, Table 1). The overall fold and structure of HsCBS complexed with AOAA (Fig. 6A) was found largely similar to the basal conformation of HsCBS reported previously [21, 28]. Forced modeling of PLP-AOAA adduct into both polypeptide chains repeatedly resulted in close contacts and strained conformation. Refinement and iterative model building eventually yielded structure, in which observed electron densities correspond to PLP bound to K119 in chain A (Fig. 6B) and PLP-AOAA adduct in chain B (Fig. 6C). In chain A, the PLP cofactor binds a conserved K119 residue via a Schiff base bond, thus forming internal aldimine, which is stabilized in the catalytic cavity by multiple hydrogen-bonding interactions with the surrounding residues. Specifically, hydrogen bonding between the S349 and the pyridine ring as well as S147 and N149 and of hydroxyl group of the PLP stabilizes the cofactor seating in the catalytic pocket. Residues T257, G258 and T260 anchor the PLP through an extended hydrogen bonding network with the phosphate moiety (Fig. 6D).

The presence of the PLP-AOAA adduct induced a rearrangement of the amino acids involved in the stabilization of the cofactor (Fig. 6E). Although the linkage between PLP and K119 is broken, the residue remains in close proximity and participates in the stabilization of the phosphate moiety. The AOAA moiety of the PLP-AOAA oxime intermediate extends toward the entrance to the catalytic cavity and is stabilized by a new network of hydrogen bonds with residues T146, T150 and Q222 in addition to S147 and N149 residues involved in stabilization of the K119-PLP internal aldimine (Fig. 6D).

To better understand the structural difference between binding of the inhibitor AOAA and the canonical substrate Ser to HsCBS, we modeled the Ser-PLP intermediate into HsCBS based on the crystal structure of such intermediate in tScCBS (Fig. 6F based on PDB# 6C2Q [18]). Similarly to AOAA, the Ser moiety of Ser-PLP intermediate extends toward the entrance of the catalytic center and is stabilized by a hydrogen bonding network consisting of the same residues (T146, S147, T150 and Q222). More importantly, residues G305 and Y308 are involved in a hydrogen bonding network with the hydroxyl of Ser further stabilizing the intermediate in the catalytic cavity compared to PLP-AOAA. This additional interaction is crucial for the catalytic cycle as Y308 activates the beta-hydroxyl leaving group of Ser and thus facilitates conversion of PLP-Ser into an aminoacrylate intermediate.

Taken together, our crystal structure of HsCBS with PLP-AOAA adduct together with a model of PLP-Ser intermediate provide new molecular details about mechanism underlying AOAA inhibitory action on CBS and may be useful in the characterization and design of future CBS inhibitors.

## Discussion

In this study, we characterized the spectral, functional and structural consequences of CBS inhibition by AOAA. AOAA has been viewed as an irreversible inhibitor of CBS (as well as other PLP-dependent enzymes), which is turned over into a dead-end external aldimine complex featuring an oxime bond [35, 36]. Indeed, AOAA is better viewed as a generic inhibitor of PLP-dependent aminotransferases rather than a CBS inhibitor [37]. In fact, it was found that AOAA is ~ 8-times more potent inhibiting human recombinant CSE than CBS in vitro [38]. Due to the lack of potent and cell-permeable CBS-selective inhibitors, AOAA remains widely used and the most potent pharmacological agent to inhibit CBS in cell culture and experimental animals to date [2].

In contrast to the irreversible nature of inhibition of PLP-dependent enzymes by AOAA [39], our data show that AOAA-inhibited CBS can be rescued by its canonical substrate Ser, but not the alternative substrate Cys (Figs. 2, 4 and 5). The formation of a PLP-AOAA intermediate is quite straightforward and well supported by our data. As Fig. 1B, C illustrates, PLP of tScCBS directly progresses from internal aldimine with residue K53 to forming an external aldimine in the presence of AOAA featuring oxime bond (Fig. 1A). Although our spectroscopic analysis is clear about the progression from PLP-AOAA to PLP-aminoacrylate intermediate in the presence of Ser (Fig. 2) and thus rescue of the catalytic activity of the enzyme (Fig. 4), the underlying molecular mechanism remains to be further characterized. Inhibition of tScCBS with a hydrazine-type inhibitor also revealed an unexpected mechanism [18]. Based on the initial spectroscopic studies and published results on other hydrazine-type inhibitors on PLP enzymes (for example the dihydroxyphenylalanine decarboxylase inhibitor carbidopa [40]), formation of a PLP-hydrazone complex inhibiting tScCBS was anticipated [18]. Instead, however, the crystal structure of tScCBS in the presence of the hydrazine inhibitor showed enzyme-bound PMP, another dead-end product responsible for tScCBS inhibition. PMP-enzyme is a common intermediate in a ping-pong catalytic mechanism of PLP enzymes, such as aspartate aminotransferase (AAT) [41]. In the forward reaction, AAT reacts with aspartate to generate oxalacetate and PMP-AAT, which reacts with alpha-ketoglutarate yielding glutamate in the reverse reaction, thus regenerating the PLP cofactor of AAT. In CBS, release of the product and regeneration of the enzyme’s internal aldimine following β-replacement mechanism bypasses the PMP intermediate. Under the β-elimination mechanism, CBS is able to proceed from external aldimine through a ketamine intermediate, releasing an α-keto product and forming PMP-CBS. However, the rate of pyruvate formation from Ser (or Cys) in the absence of Hcy is quite negligible [4]. Therefore, a plausible reaction mechanism explaining our observations could be the release of AOAA adduct over time in the presence of Ser, which may act as a required facilitator of the process, followed by a formation of internal aldimine (Fig. 1A) or indirectly through a PMP-CBS intermediate.

Structural changes associated with CBS inhibition by AOAA are quite small. The 222/208 ellipticity ratio is used to distinguish coiled-coil (≥ 1.0) from non-interacting helices (0.8–0.9) [42]. Therefore, our CD analysis suggested that non-interacting helices are more prevalent in tScCBS complexed with AOAA compared to coiled-coil helices in tScCBS complexed with Ser or after the rescue of the AOAA-inhibited enzyme with Ser (Fig. 2). Indeed, unlike in the case of PLP-AOAA oxime type external aldimine, the PLP-Ser external aldimine of HsCBS formed a richer network of hydrogen bonds, particularly with G305 and Y308, which poised this complex toward the formation of PLP-aminoacrylate intermediate and thus continuation of the catalytic cycle (Fig. 6).

**Fig. 7:**
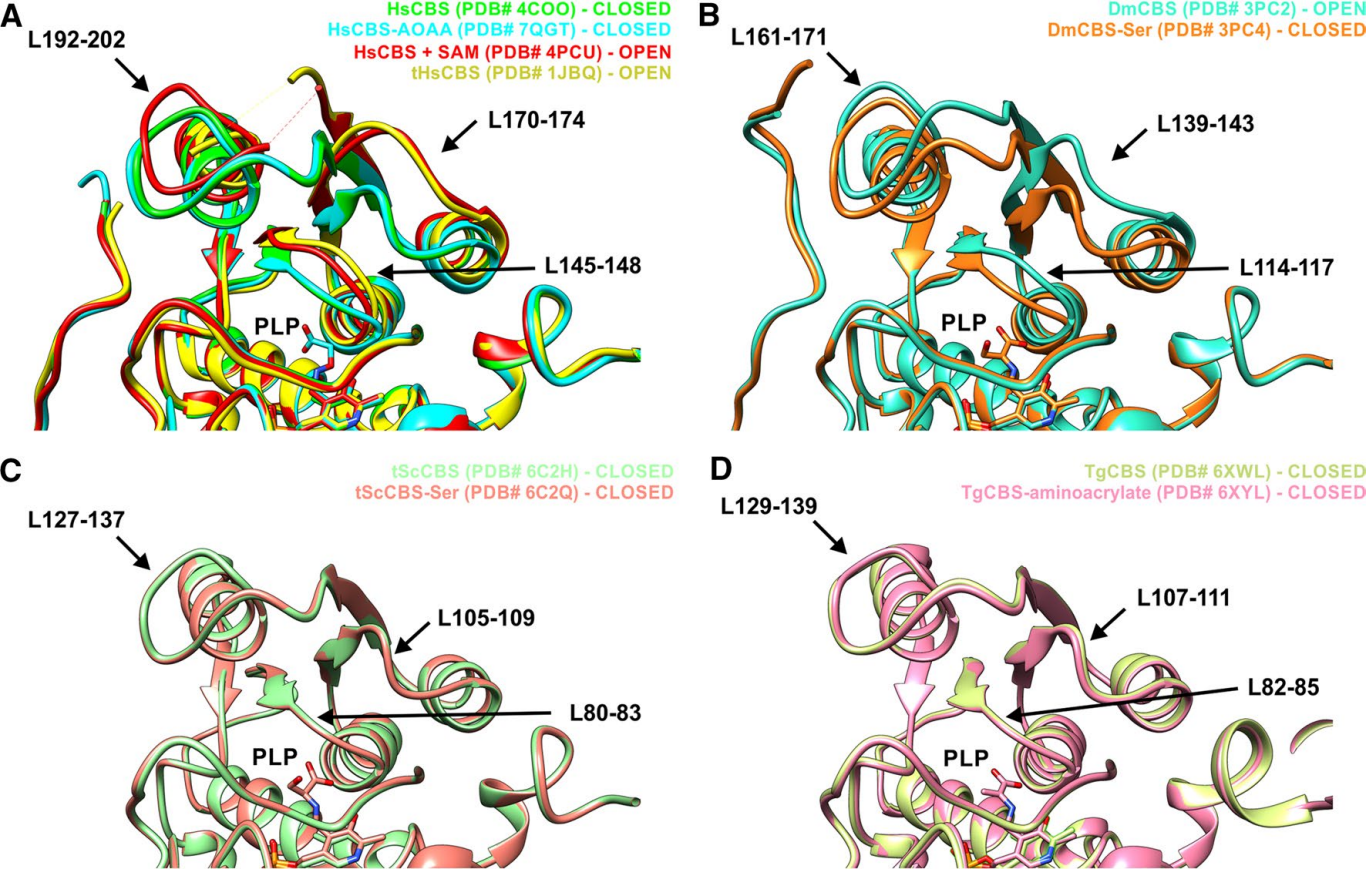
Structural comparison of the entrance to the catalytic cavity of CBS enzymes. **A** Loops L145-148, L170-174 and L192-202 can adopt collapsed (closed) conformation in HsCBS and HsCBS complexed with AOAA. However, the same loops remain relaxed (open) in tHsCBS lacking the regulatory domain or HsCBS in complex with its allosteric activator SAM. **B** The respective loops in DmCBS are in the open conformation and collapse upon binding of the substrate. **C**, **D**) The respective loops in tScCBS and TgCBS show only the closed conformation in both *apo* enzymes and enzymes with substrates bound to PLP

In addition, binding of the substrate can have different consequences in CBS enzymes. Our structure of HsCBS in complex with AOAA is the first structure of human CBS enzyme with a bound substrate (or inhibitor targeting the catalytic PLP cofactor in similar fashion as the substrates), thus allowing the assessment of the conformation of the loops delineating the entrance to the catalytic cavity upon substrate/inhibitor binding (Fig. 7). Conformation of the loops L145-148, L170-174 and L192-202 in HsCBS complexed with AOAA is very similar to those in HsCBS, i.e. the collapsed (closed) conformation, suggesting that the inhibitor (and likely the substrate as well) does not induce further changes in these loops (Fig. 7A). Interestingly, both removal of the regulatory domain and binding of SAM induce the opening of the catalytic cavity by relaxing the loops into an open conformation, which explains higher specific activity of HsCBS catalytic core and SAM-activated HsCBS compared to the basal status of HsCBS in the absence of SAM [20, 43, 44]. Similarly, the conformation of the respective loops was found open in a highly active DmCBS and closed when bound to Ser or forming aminoacrylate intermediates (Fig. 7B) [32]. However, no conformational change of the respective loops upon substrate binding was detected for tScCBS (Fig. 7C) [18] and *Toxoplasma gondii* TgCBS (Fig. 7D) [23]. Therefore, it seems that the conformation of the loops delineating catalytic cavity is not a universal feature as it differs among CBS enzymes with available structural information. It likely depends on multiple factors, such as presence of the heme cofactor or the ability to bind/respond to SAM.

Despite a lot of effort invested into discovery of novel, potent and selective CBS inhibitor in the past decade, AOAA remains the most commonly used CBS inhibitor to date [2]. Efficacy of AOAA was often found to be cell-dependent with primary cells like human skin fibroblasts being very sensitive with 3 µM AOAA shutting down CBS-derived H_2_S biogenesis [12], while most transformed cells like colon cancer cells required much higher concentrations of 0.1–2 mM AOAA to achieve similar level of inhibition [9, 15, 45]. Here we showed that 150 µM AOAA achieved ~ 90% inhibition of H_2_S production of HEK293 cells (Fig. 5A, B), which also translated to decreased cellular bioenergetics (Fig. 5E–J). Supplementation of growth medium with 10 mM Ser resulted in a significant ~ 29% reduction of cellular H_2_S productivity (Fig. 5A, B) suggesting that a shift in balance between the canonical Ser and the alternative Cys substrate of CBS leading to H_2_S production by itself has a profound impact on overall H_2_S producing capacity of HEK293 cells. More importantly, supplemental Ser led to catalytic re-activation of AOAA-inhibited CBS activity and partially rescued H_2_S-producing capacity of HEK293 cells (Fig. 5A, B) as well as normalized cellular bioenergetics (Fig. 5E–J). These data strongly corroborate our spectral and biochemical observations and together provide high confidence that the efficacy of AOAA as inhibitor of CBS in cellular or in vivo context might be impaired or compromised by Ser content. Considering the results presented here, the differences in efficacy of AOAA inhibiting CBS could plausibly be explained by changes in cell intermediary metabolism, availability and concentrations of Ser and Cys competing on CBS and ultimately, ability of Ser, but not Cys, to reactivate AOAA-inhibited CBS. Specifically, many cancer cells, including colon cancer cells HCT116, were found highly dependent on Ser supplementation and/or de novo Ser synthesis for proliferation [46] suggesting that these cells may maintain high intracellular concentrations of Ser. Indeed, HCT116 cells maintain high ~ 1.7 mM intracellular Ser concentration, which allosterically activates pyruvate kinase M2 isoform and thus controls glycolytic flux and supports cancerous metabolic reprograming [47]. On the contrary, depleted plasma and presumably also tissue levels of Ser and elevated levels of Cys were found in samples from DS individuals [48, 49]. Thus, cellular metabolic rate and Ser/Cys ratio likely represent the key factors determining the efficacy of AOAA inhibiting CBS-derived H_2_S biogenesis. Previously, we found that the Ser/Cys ratio is the main determinant of H_2_S production by CBS in vivo [5]. Together, intracellular concentrations, availability and compartmentalization of competing CBS substrates Ser and Cys play the critical regulatory role in determining H_2_S-producing capacity and its response to pharmacological inhibition.

Nevertheless, it has to be noted that both Ser and AOAA has many cellular targets and thus likely affect metabolism and bioenergetics independently of CBS/H_2_S axis as well. Serine is the main source of one-carbon (1C) units, which are required for a variety of fundamental cellular activities, such as nucleotide synthesis, redox homeostasis and epigenetic maintenance [50]. Although de novo Ser synthesis sufficiently sustains a rapid cell proliferation [51], extracellular Ser could promote anabolic pathways and bioenergetics via stimulating the mammalian target of rapamycin (mTOR) signaling pathway [50]. Impact of AOAA on mitochondrial function is even more complex due to lack of its selectivity, which we reviewed recently elsewhere [2]. Briefly, AOAA has been shown to inhibit other enzymatic sources of H_2_S, namely CSE and Cys aminotransferase CAT. In addition, AOAA inhibits glutamic oxaloacetic transaminases GOT1 and GOT2, key players in the malate/aspartate shuttle, which transfers electrons generated in cytoplasmic glycolysis to support mitochondrial oxidative phosphorylation. Lastly, AOAA inhibits alanine aminotransferase ALT converting glutamate into α-ketoglutarate, which feeds into the Krebs cycle and promotes mitochondrial respiration. Previous study showed that mitochondrial dysfunction remodels 1C metabolism in human cells through activating transcription factor 4 (ATF4)-mediated increase in de novo Ser biosynthesis and transsulfuration including H_2_S production [52]. Thus, our data showing that serine reactivated AOAA-inhibited CBS rescued cellular H_2_S production and mitochondrial function support and essentially confirm the connection between 1C metabolism and bioenergetics via CBS/H_2_S axis.

In conclusion, here we demonstrated that AOAA covalently binds to HsCBS forming external aldimine featuring oxime bond, which leads to inhibition of CBS activity. However, in contrast to an assumption about the irreversible nature of CBS inhibition by AOAA, we showed that the canonical CBS substrate Ser, but not alternative substrate Cys leading to H_2_S biogenesis, can rescue CBS activity. This study underscores the complexities of AOAA as a CBS-specific inhibitor and predicts that the activity of AOAA in the cellular environment (in health or in disease) may be substantially modulated by the biochemical environment (i.e. by the concentration of Ser). In addition, since the PLP cofactor was recovered by Ser from a presumably dead-end PLP-AOAA complex, based on our findings, we conclude that targeting PLP in search of novel CBS inhibitors may not necessarily yield an irreversible or suicide inhibitor, but rather may be at least partially reversible, depending on the cellular environment. We hypothesize that future in silico modeling work (based, at least in part, on the structure and mechanisms outlined in this report) could be utilized to yield potentially novel classes of CBS-specific inhibitors targeting structurally-specific features of HsCBS, such as conformational flexibility of the loops delineating catalytic cavity or allosteric mechanism underlying CBS activation by SAM.

## Data Availability

The atomic coordinates and structure factors of the engineered human CBSΔ516-525 complexed with aminooxyacetic acid inhibitor has been deposited in the Protein Data Bank with accession code 7QGT.
